# Limonene Derivative of Spherosilicate as a Polylactide Modifier for Applications in 3D Printing Technology

**DOI:** 10.3390/molecules25245882

**Published:** 2020-12-12

**Authors:** Dariusz Brząkalski, Bogna Sztorch, Miłosz Frydrych, Daria Pakuła, Kamil Dydek, Rafał Kozera, Anna Boczkowska, Bogdan Marciniec, Robert E. Przekop

**Affiliations:** 1 Faculty of Chemistry, Adam Mickiewicz University in Poznań, 8 Uniwersytetu Poznańskiego, 61-614 Poznań, Poland; dariusz.brzakalski@amu.edu.pl (D.B.); frydrych@amu.edu.pl (M.F.); Darpak@amu.edu.pl (D.P.); 2 Centre for Advanced Technologies, Adam Mickiewicz University in Poznań, 10 Uniwersytetu Poznańskiego, 61-614 Poznań, Poland; bogna.sztorch@amu.edu.pl; 3 Faculty of Materials Science and Engineering, Warsaw University of Technology, 141 Wołoska, 02-507 Warsaw, Poland; kamil.dydek@pw.edu.pl (K.D.); anna.boczkowska@pw.edu.pl (A.B.); 4 Technology Partners Foundation, 5A Adolfa Pawińskiego, 02-106 Warsaw, Poland; rafal.kozera@technologypartners.pl

**Keywords:** spherosilicate, limonene, hydrosilylation, polylactide, 3D printing, FDM, FFF, injection moulding, rheology, thermal analysis

## Abstract

The first report of using limonene derivative of a spherosilicate as a modifier of polylactide used for 3D printing and injection moulding is presented. The paper presents the use of limonene-functionalized spherosilicate derivative as a functional additive. The study compared the material characteristics of polylactide modified with SS-Limonene (0.25–5.0% *w*/*w*) processed with traditional injection moulding and 3D printing (FFF, FDM). A significant improvement in the processing properties concerning rheology, inter-layer adhesion, and mechanical properties was achieved, which translated into the quality of the print and reduction of waste production. Moreover, the paper describes the elementary stages of thermal transformations of the obtained hybrid systems.

## 1. Introduction

Three-dimensional (3D) printing is one of the most dynamically developing modern technologies. It was discovered and patented in the 1980s by Charles Hull (SLA technique) and was protected by a patent for 20 years [1]. The development and growth of interest in additive technologies has been going on continuously for about 15 years and is mainly caused by the expiration of patents, but also a decrease in printer prices and their increased availability. At that time, technological solutions became commercially available, initially only to the largest enterprises, now it is increasingly used by the sector of small and medium-sized enterprises, but also by individual consumers. Additive technologies are among those of much demand constituting the main pillar of Industry 4.0 [2]. Initially, 3D printing technology did not arouse much interest, and in the Gartner report from 2012 it was included in the area of the so-called ‘Trough of Disillusionment’ [3]. However, the aforementioned development of new techniques was so rapid that, in 2013, the position of the 3D printing sector changed dramatically and in 2015 Gartner published a separate report dedicated to it [4]. Additive technologies can be divided into: photochemical DLP [5], SLA [6,7], laser SLS [8], thermal FDM [9] or LOM [10].

One of the most popular additive techniques is FDM (fused deposition modelling). The technology originally developed by Stratasys involves extrusion from a die heated above the polymer melting point and then applying it layer by layer in the direction of the Z axis [11]. The extruded material is in the form of a filament with a diameter of usually 1.75 mm. The advantages of the FDM method are its versatility and accessibility [12], as well as the ease of designing and making a model of any shape and geometry [13]. The disadvantages include, first of all, insufficient mechanical strength in the direction of the Z axis due to the appearance of air gaps between successively superimposed layers [14]. This effect does not occur in the case of other traditional methods, such as injection moulding, where solid objects are obtained.

Such air gaps can contribute to the appearance and initiation of cracks and material design defects. Therefore, attempts are continuously made to reduce poor quality of printed materials and improve the interfacial strength of printed models [15]. In addition, when compared to the traditional method such as injection moulding, 3D printing generates a number of technological problems related to insufficient process speed [16] or product quality (often requiring additional post-processing) [17].

Significant disadvantages hampering the future of 3D printing also include high waste generation, higher than in traditional techniques such as extrusion or injection moulding. An attempt to eliminate unfavourable features by improving the processing device, which is the printer, have encountered significant limitations. In the FDM technique, several thermoplastics are usually used with the greatest emphasis on such polymers/copolymers as: PLA, PA, ABS, TPU. The same types of plastics are mostly used in 3D printing and mature processing techniques. FDM is micro-processing and has significant differences when compared to classic thermoplastics processing techniques, such as lower extruder pressure or smaller cross-sections of the canals in which the molten material flows. Therefore, plastics dedicated for 3D printing should be designed to show the properties addressing these differences (e.g., higher MFI/lower viscosity).

In the FDM technique, polylactide is the most commonly used polymer, mainly due to its ease of its processing, low thermal shrinkage [18] and biodegradability. Degradation rate is low enough to make it resistant to mild weather conditions [19] which is why, next to ABS, it is most often used in medicine [20]. The melting point of PLA is around 150–170 °C and is lower than those of many other popular polymers, it also requires much less energy due to low heat of fusion, so it can be widely used in various processing techniques [21]. One of the disadvantages of PLA limiting its application is low mechanical strength, especially the impact resistance [22]. In order to improve these parameters, structural fillers and plasticizers have been used, e.g., glass and carbon fibres, ceramic or metallic fillers, and glycols [23].

Chemical modifiers that improve the functional properties of composites, such as organic and organosilicon compounds, can also be applied. Limonene (4-isopropenyl-1-methylcyclohexene)—is the main component of oils obtained from waste citrus peels (biomass). It can be obtained by natural and synthetic methods, e.g., using pyrolytic processes [24]. Simple distillation or steam distillation of citrus peels makes it possible to recycle the waste citrus peels from food industry and, at the same time, to obtain pure limonene with a small amount of toxic waste. Annually, these methods produce over 70,000 tons of this compound. In 3D printing technology, it is used as a solvent for support materials made of high impact polystyrene [25]. From the point of view of synthetic applications, limonene should be classified as a green olefin that is subjectable to hydrosilylation reaction [26]. The use of limonene as a building block is one of the many necessary steps in creating a chain of products based on raw materials of natural origin with a lower environmental impact. In its pure form, however, it has a low boiling point for PLA processing (176 °C), which may cause its boiling during processing and, as a result, introducing gases into the polymer, as well as emission of vapours to the environment. Nevertheless, attempts have been made to use it in the processing of PLA, when it showed a plasticizing effect on the polymer matrix, which is a great advantage due to the brittleness of the neat polymer [27].

Polyhedral oligomeric silsesquioxanes are well-known organosilicon compounds, mostly recognized for their high symmetry, excellent solubility, or unparalleled simplicity of synthetic protocols [28]. Among those, a subgroup called spherosilicates may be distinguished, sometimes identified as a different group of compounds, and in such case, both spherosilicates and silsesquioxanes are collectively called cage siloxanes [29]. Due to their high thermal stability and good dispersion properties, they are considered interesting functional additives for polymer processing. In our previous works we have presented approaches towards processing of low-density polyethylene (LDPE) with polyhedral oligomeric silsesquioxanes [30], as well as spherosilicates [31]. These studies allowed determination of the critical level of practical loading in the LDPE matrix, which was much lower than what can be found in numerous literature reports, and was parallelly confirmed by Romo-Uribe et al. [32,33,34]. One derivative from among the tested ones was found to be particularly interesting, it was SS-Limonene, a product of limonene hydrosilylation with Octahydrospherosilicate. The results suggested its mildly plasticizing effect on the polymer matrix, besides its content improved thermal and mechanical properties of the obtained materials. Therefore, in this study, we decided to assess the applicability of this derivative as a functional and processing additive for PLA for 3D printing.

## 2. Results and Discussion

### 2.1. Characterization of SS-Limonene (1,3,5,7,9,11,13,15-octa(Dimethyl((2-(4-methylcyclohex-3-en-1-yl)propyl)silyl)-Pentacyclo [9.5.1.1^3,9^.1^5,15^.1^7,13^ ]Octasiloxane

**Figure 1:**
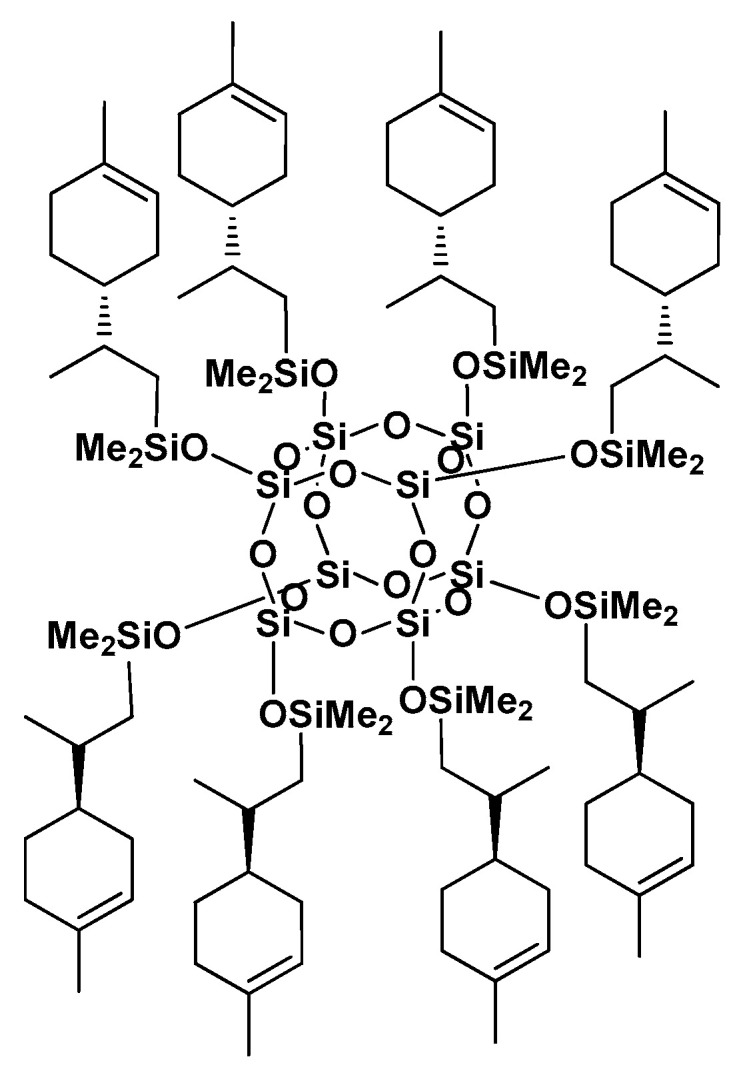
Structure of SS-Limonene.

SS-Limonene (Figure 1) was prepared according to the synthesis procedure described in Section 3.3 and the reaction completion was determined by FT-IR spectra analysis, the disappearance of the characteristic signals assigned to stretching and bending vibrations of the Si-H group was observed at 2141 and 889 cm^−1^, respectively). Upon completion of the reaction, the hydrosilylation reached ~99% conversion. The structure and purity of the modifier was confirmed by NMR and MALDI-TOF-MS analyses.

The purity and chemical structure of the synthesized compound was confirmed by NMR spectroscopy, with the following signals assignment:

^1^H-NMR (400 MHz, CDCl_3_): δ (ppm) = 5.36 (s, 8H, ring position 3), 2.00–1.87 (m, 24 H, ring positions 1, 2, 5), 1.78–1.56 (m, 24 H, ring positions 2, 5, =CH- isopropenyl methylidene), 1.63 (s, 24 H, -CH_3_ methyl attached to ring position 4), 1.34–1.18 (m, 16 H, ring position 6), 0.90 (d, 24 H, isopropenyl methyl), 0.75–0.69 (m, 8H, isopropenyl -CH_a_H_b_-), 0.52–0.44 (m, 8H, isopropenyl -CH_a_H_b_-), 0.15 (s, 48H, SiMe_2_); ^13^C-NMR (101 MHz, CDCl_3_): δ (ppm) = 133.96, 121.28, 121.26, 41.48, 41.33, 33.08, 32.93, 31.19, 31.12, 28.80, 28.24, 26.71, 25.71, 23.64, 23.63, 22.87, 22.48, 19.52, 19.22, 0.83, 0.72, 0.66; ^29^Si-NMR (79,5 MHz, CDCl_3_): δ (ppm) = 12.78 (SiMe_2_), −109.10 (core). IR (ATR): 2980–2867, 1252, 1169–1069, 869–734, 549. MALDI-TOF-MS: [M + Na]^+^: 2127.9581 (calc.), 2127.9606 (anal.)

### 2.2. Density of SS-Limonene/PLA Blends and Mass/Quantity Waste Factor of the Printed Samples

Densities of all samples were measured by the hydrostatic method. Measurements were performed for the samples of 1 cm in length. The average densities of all samples and the base sample of neat PLA were at the same level of around 1.24 g/cm^3^. The waste factor of printed samples was also analysed for the bars printed with 100% infill. Data are collected in Table 1. On the basis of the obtained data, the mass and the volume waste factors were determined according to the formula:

(1) $$Wf\left[m,q\right]=\frac{mass\;\left(quantity\right)of\;corect\;samples\times 100\%}{mass\;\left(quantity\right)\;of\;total\;samples}$$

The calculated waste factors clearly showed that with decreasing content of the SS-Limonene modifier, the amount of 3D printing waste decreases. The issues with printing the samples with high loading of SS-Limonene can be explained on the basis of the additive polymerizing and curing during the polymer processing, which is further explained in Section 2.4 and Section 2.5.

### 2.3. Rheology

**Figure 2:**
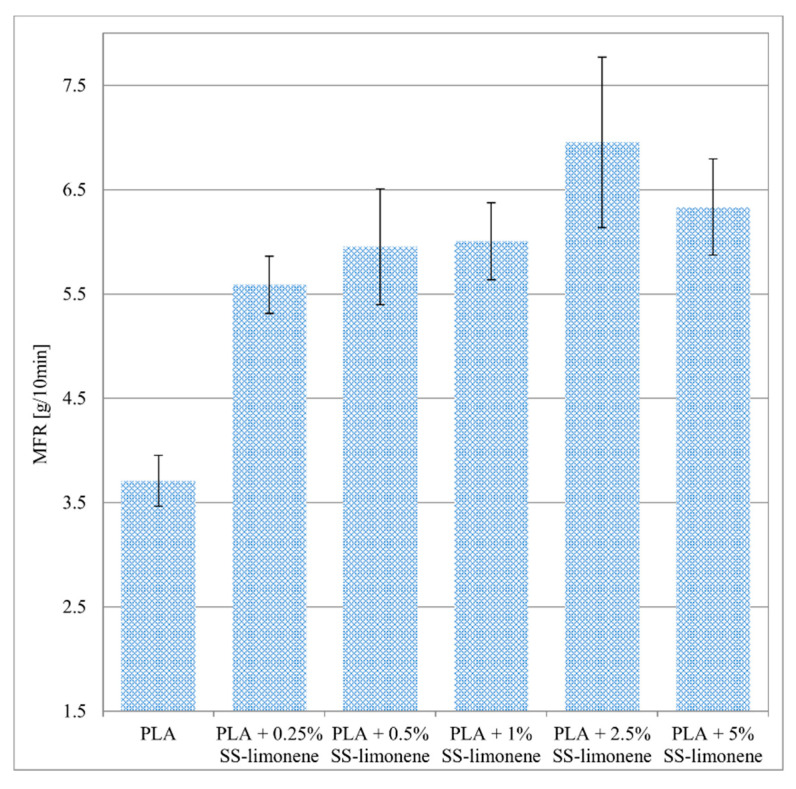
Result of MFR measurements.

The melt flow index (MFI) of pure PLA at 190 °C is 3.7075 g/10 min (Figure 2). For the PLA composites with SS-Limonene the MFI value increased with increasing concentrations of the filler. The composite samples containing 2.5% concentration of the additive are characterized by slightly higher MFR value. On the basis of the data analysis, it can be concluded that SS-Limonene as an additive to the polymer matrix will have a positive effect on its processing.

**Figure 3:**
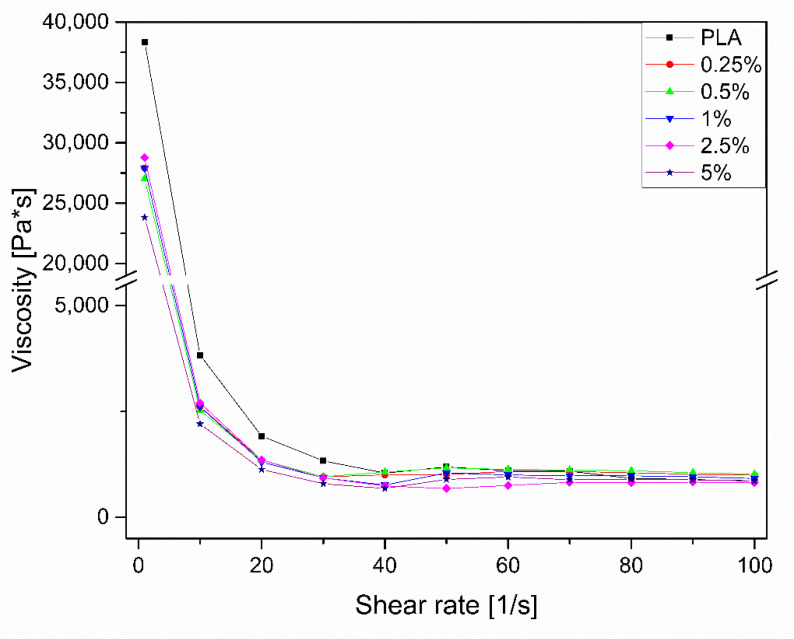
Viscosity measurement results.

A capillary rheometer was used to determine the relation between shear rate and viscosity (Figure 3). At low shear rates, the viscosities of all compositions are lower than those of neat PLA, as a consequence of SS-Limonene acting as a plasticizer.

### 2.4. Microscopy

**Figure 4:**
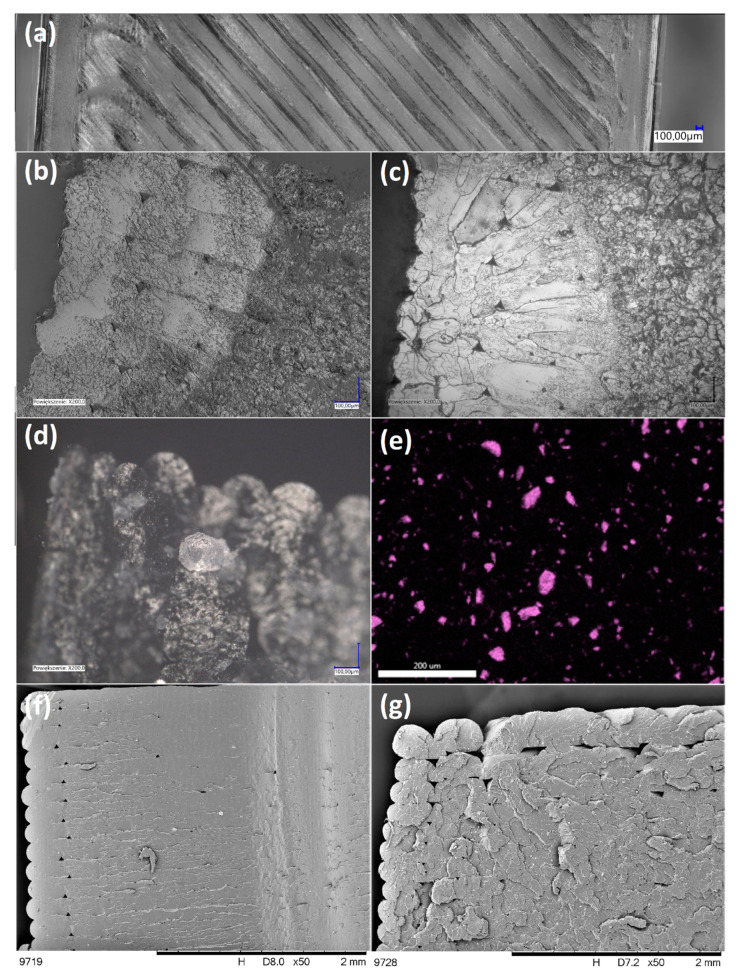
Optical microscopic images (**a**–**d**) and SEM images (**e**–**g**) of printed samples: outer surface (**a**), fractured sample from neat PLA (**b**), fractured sample from 0.25% SS-Limonene/PLA (**c**), a crystal of polymerized SS-Limonene (**d**), silicon EDS image of 0.25% SS-Limonene/PLA fractured sample (**e**), fractured sample from 0.25% SS-Limonene/PLA (**f**), fractured sample from 2.5% SS-Limonene/PLA (**g**).

**Figure 5:**
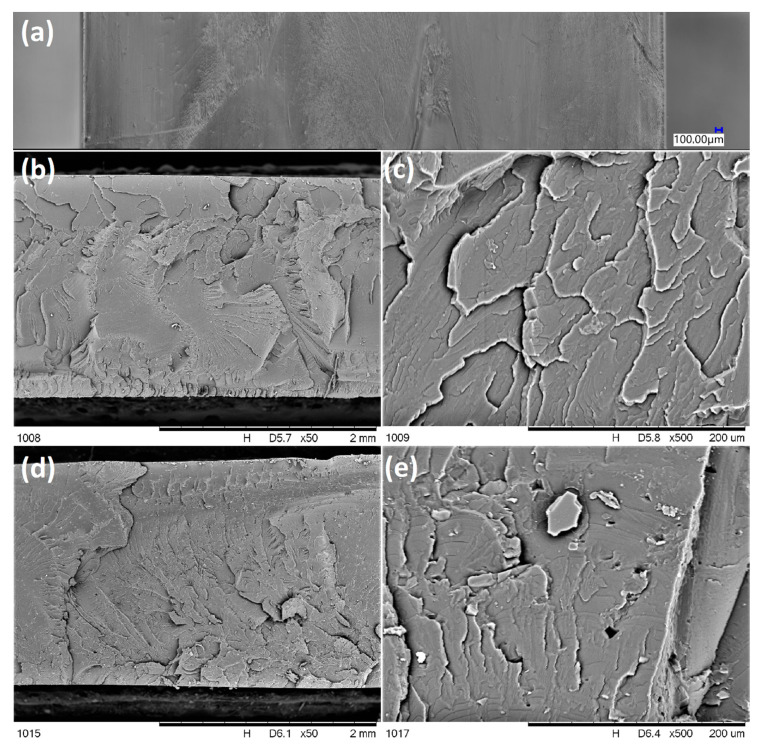
Optical microscopic image (**a**) and SEM images (**b**–**e**) of injection moulded samples: outer surface (**a**), fractured sample from neat PLA (**b**,**c**), fractured sample from 2.5% SS-Limonene/PLA (**d**,**e**).

Figure 4 and Figure 5 present the outer and inner texture of 3D-printed (Figure 4) and injection-moulded samples (Figure 5) under a digital light microscope and SEM with an additional EDS analysis (Figure 4e). For the printed samples, different morphologies of the outer and inner regions of the fractured sample (Figure 4b,c) can be seen due to the printing pattern, as the infill has a grid pattern for improved mechanical strength, while the outer layers are made in a rectangular pattern and therefore can be seen as perpendicular to the fracture plane. This is a result of the standard printing process conditions, where the 3D printer outlines the outer shapes of the sample with straight lines first, and then infills the object with a chosen pattern. However, the outline of the outer layers is, in fact, of the highest interest, as it allows observation of the inter-layer interfaces between the individual extrudate strands (and, on this basis, an assessment of the layer-to-layer bonding), as well as the size of the air gaps. In Figure 4b, the inter-layer interfaces between extrudate strands of the neat PLA sample can be clearly seen, which contributes to the low mechanical resistance of the printed PLA objects. On the other hand, for 0.25% SS-Limonene/PLA sample (Figure 4c), this interface is hardly visible, which explains the improved inter-layer adhesion and mechanical rigidity of the samples, which made them mechanically more similar to the injection-moulded ones. However, the formation of air gaps was rather unaffected. Also, on closer inspection, all the samples containing SS-Limonene additive (either 3D printed or injection moulded) contained particles visible both under the light microscope (Figure 4d) and SEM (Figure 4f,g and Figure 5d,e). EDS analysis of silicon allowed identification of the particles to be agglomerates of the polymerized additive (Figure 4e). With increasing loading, more agglomerates of such particles were visible, which contributed to the issues with printing the samples containing high amounts of SS-Limonene. Polymerization of the additive is further discussed in Section 2.5. Additionally, for injection-moulded samples, together with the mentioned particles, small air gaps were observed (Figure 5d,e), which are not present in the sample made from neat PLA (Figure 5b,c). It may be due to difficulties with degassing of the polymer melt, or the generation of gas products either of evaporation or decomposition of SS-Limonene, as the thermogravimetric analysis thereof under the PLA processing temperatures revealed a small mass loss.

### 2.5. Thermal Analysis Results

Thermal effects for SS-Limonene/PLA compositions were measured by differential scanning calorimetry (DSC) and thermogravimetric analysis (TGA).

**Figure 6:**
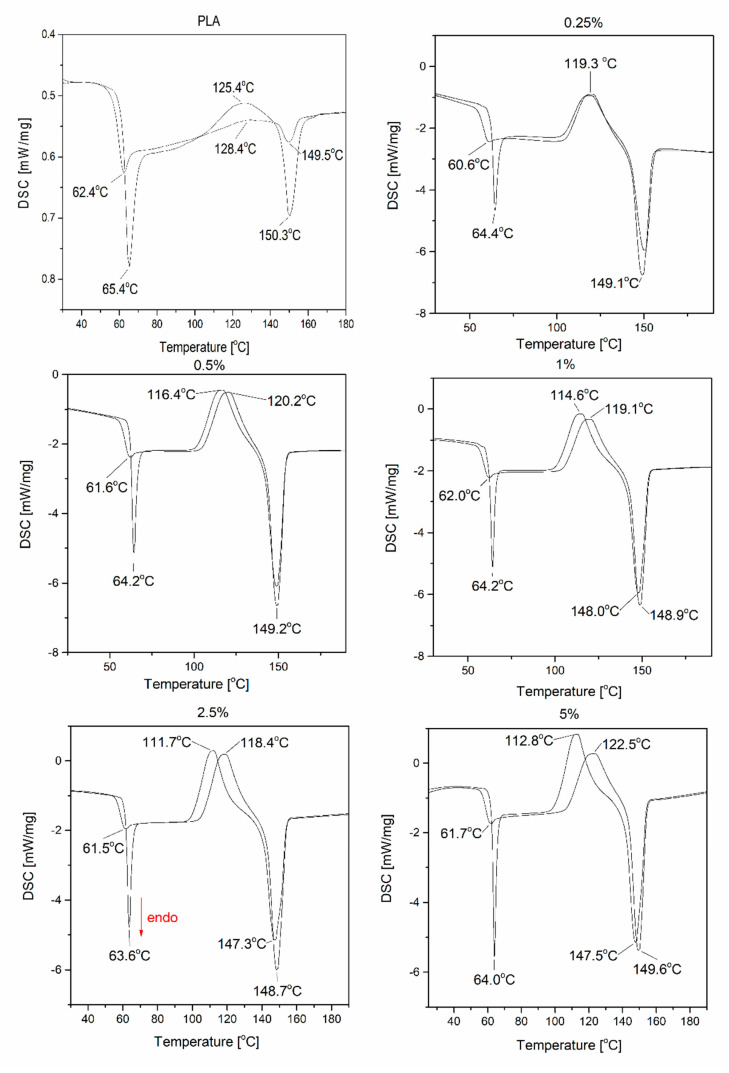
DSC curves recorded for samples of SS-Limonene/PLA composite.

DSC analysis was performed to determine the effect of SS-Limonene addition on the glass transition (T_g_), crystallization (T_c_) and melting (T_m_) temperatures of the composites. The graphs are shown in Figure 6. In each case, a large peak of glass transition is noticeable at the first heating cycle, which is related to the low crystallinity of the extruded samples (high proportion of the amorphous phase), due to the rapid cooling of the polymer (in air and in a cooling bath during extrusion). The glass transition temperature (T_g_) after the second heating cycle of the test samples containing the organosilicon additive is shifted towards lower values relative to that observed for the neat PLA. This is due to the plasticization of the polymer matrix. Similar conclusions can be drawn in the context of T_cc_, as the presence of the plasticizing phase increases the freedom of the chains in the amorphous phase, accelerating the initiation of crystallization. The change in T_m_ and T_cc_ values also shows that SS-Limonene is at least partially mixed with PLA and the interaction of these two components occurs, despite the presence of the polymerized additive phase visible in the microscopic photos (SEM-EDS, light microscopy). On the basis of T_g_, the strongest plasticizing effect was observed for the system containing 0.25% of the above-mentioned modifier, but the lowest melting point was observed for the system containing 5% of SS-Limonene. The DSC analysis of the modifier allowed observation of the polymerization of the compound on heating of the sample, which was also confirmed by microscopic analysis as an effect of agglomeration of the polymerized additive in the PLA matrix. A similar effect has been observed in our earlier work, however, most likely due to lower processing temperatures of LDPE, the agglomeration was far less severe [31].

**Figure 7:**
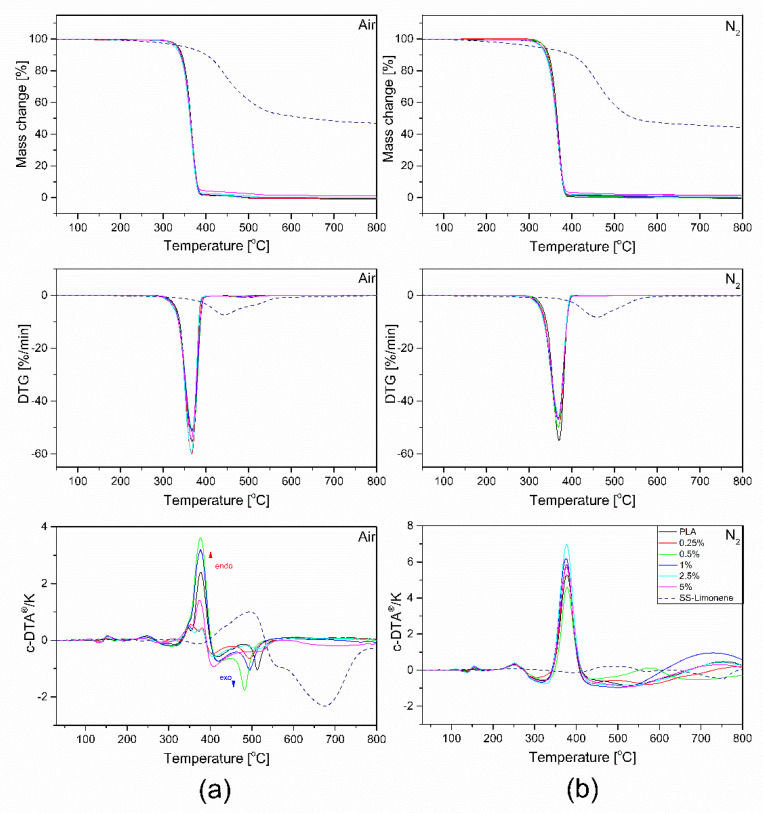
TGA, c-DTA and DTG curves of SS-Limonene/PLA composite in (**a**) air and (**b**) nitrogen atmosphere.

All results of the TGA, DTG and c-DTA measurements are presented in Figure 7. The parameters determined, including the temperature of 1% mass loss, onset, and temperature at the maximum rate of mass loss are collected in Table 2. The process of thermal decomposition of samples was carried out in both nitrogen and air atmosphere. It should be remembered that thermal changes in thermoplastic systems at temperatures above the melting point of the matrix should not be defined as the ‘thermal resistance’ or ‘thermal strength’ of the composite, but refer to the influence of the applied additives on the thermal stability of the polymer system in molten state and the interaction between the system components in the melt. Based on the observation of the complete thermal analysis, i.e., the derivatographic curve, DTG and c-DTA results obtained both in air and nitrogen atmosphere, three stages of thermal transformation can be distinguished. The first step, observed in both air and nitrogen atmosphere, at 140–160 °C is related to the polymerization of SS-Limonene. The second stage, taking place in a nitrogen atmosphere at a temperature above 365.7 °C, is related to the cracking of PLA chains with the simultaneous endothermic distillation of mers, including SS-Limonene degradation products and lactides. According to the c-DTA curve determined in the air atmosphere, the endothermic distillation process overlaps with the exothermic oxidation process of the cracking products. In the last, third stage, in the air atmosphere, one more signal is observed at a temperature of 550–700 °C, which characterizes the process of burning coke, originating mainly from the organosilicon derivative. Stage 1 refers to the functional properties of the composite material, while stages 2 and 3 describe the irreversible processes of thermal decomposition of a mixture of molten PLA and an organosilicon derivative.

### 2.6. Contact Angle Measurements

The contact angle measurements were performed for SS-Limonene/PLA composites obtained by two different methods—FDM and injection moulding. For the measurements of 3D-printed samples, the samples were placed with the layer-by-layer deposition plane oriented parallelly to the plane of the goniometer stage. The contact angle of the neat PLA was 81.4° for the printed samples and 83.6° for the samples obtained in the injection process, in both cases the surface before modification showed hydrophilic properties. Modification of PLA with SS-Limonene increased the hydrophobicity of all the tested samples (see Table 3). For the printed samples, regardless of the modifier concentration, a hydrophobic surface effect was obtained (the value of the contact angle increased to above 90°). For the samples obtained by injection moulding, the increase in the value of the contact angle was insignificant and it remained at a similar level (max. by 4.3°). The difference in the degree of hydrophobicity of printed and injected samples is due to their surface structure and microstructure. 3D printing allows obtaining microstructures and surface roughness, which results in higher values of the contact angle. This thesis was confirmed by microscopic observations (see Section 2.4). On the other hand, injection moulding produces more smooth surfaces (if no modification of the mould surface is applied), which reduces the microstructure effect on the surface and therefore almost no effect of the organosilicon additive can be observed.

### 2.7. Mechanical Properties

#### 2.7.1. Tensile Strength and Flexural Strength

**Figure 8:**
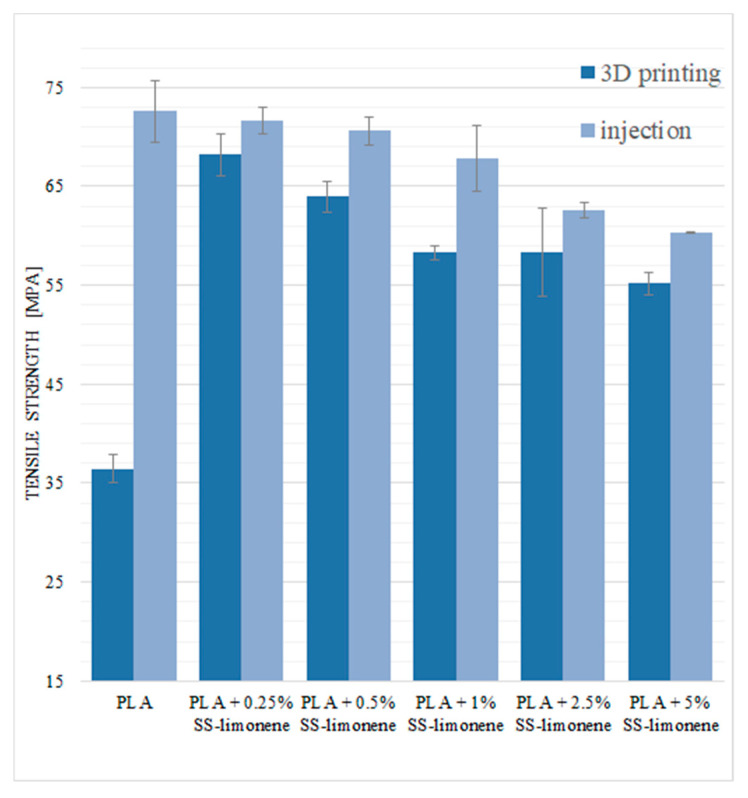
Tensile strength of SS-Limonene/PLA in 3D printing and injection moulding.

Mechanical tests were carried out for the modified samples obtained by both 3D printing and traditional injection moulding. For the 3D-printed samples, the tensile load was applied parallelly to the plane of layer-by-layer deposition. The basic tensile strength values for neat PLA are 36.5 MPa for the samples obtained by the FDM method and 72.6 MPa for the samples obtained by the injection moulding (Figure 8). This difference is due to the technique of producing the dumbbells for tests. Lower values of mechanical parameters of printed samples are mainly related to low inter-layer adhesion between the extrudate strands and the presence of air gaps between the applied layers. In the caseof injection moulding, the materials are more solid with little to no structural defects. The addition of the SS-Limonene modifier increased the tensile strength of the printed samples, which brought them closer to the injection moulded ones. The highest value was observed for PLA with the content of 0.25% of the modifier, this value decreased with increasing concentration. This is mainly due to the improved flow of the polymer as shown in the capillary rheometry analysis of the samples (Section 3.1), as well as improved inter-layer adhesion (Section 2.4), which resulted in increased material consistency and improved fusing of the print paths (extrudate strands). For all the tested samples, high values of standard deviation were obtained, which is a characteristic feature of FDM printed objects due to the mentioned structural inconsistencies.

For the samples obtained by injection moulding, the tensile stress values are the highest for pure PLA and decrease with increasing loading of the modifier. This result can be explained by two reasons: one is the plasticizing effect of SS-Limonene, and the other is the presence of discontinuities in the polymer phase introduced together with the additive, that is the polymerized SS-Limonene agglomerates and additional air micropockets, as confirmed by the microscopy.

**Figure 9:**
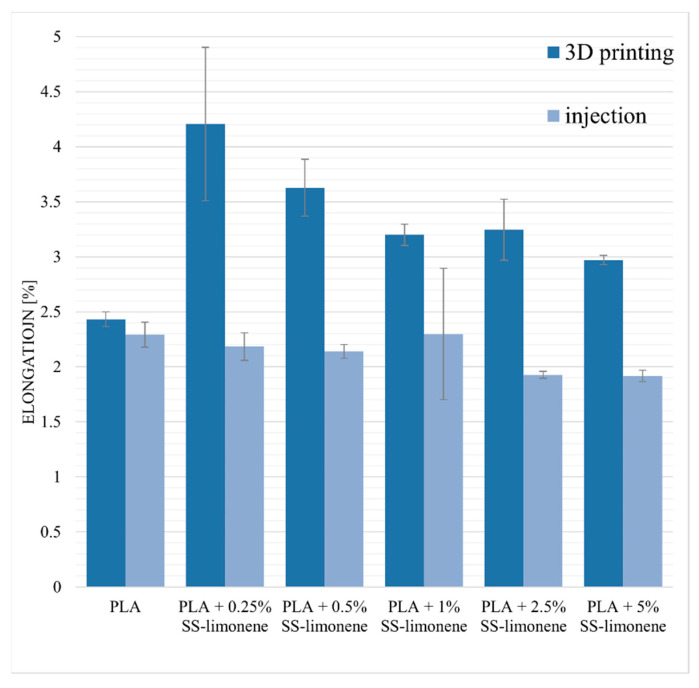
Elongation at maximum load of SS-Limonene/PLA in 3D printing and injection moulding.

The elongation at maximum load for neat PLA samples obtained by 3D printing and injection moulding is characterized by similar values (2.43% and 2.29%, Figure 9). The addition of SS-Limonene to the samples obtained by the FDM method significantly improves the plasticity of the material. Higher elongation values in the case of modified samples indicate increased “mobility” of the polymer phase as a result of plasticization by SS-Limonene [35]. The plasticizer isolates the chains and spherulites of macromolecules, reducing the interaction between them. The highest value was obtained for PLA + 0.25% SS-Limonene, which was 4.21%. At higher loadings, the effect of the additive polymerization takes over and decreases the plasticizing effect. In the case of injection-moulded samples, the addition of the modifier gave negligible effects.

#### 2.7.2. Bending Tests

**Figure 10:**
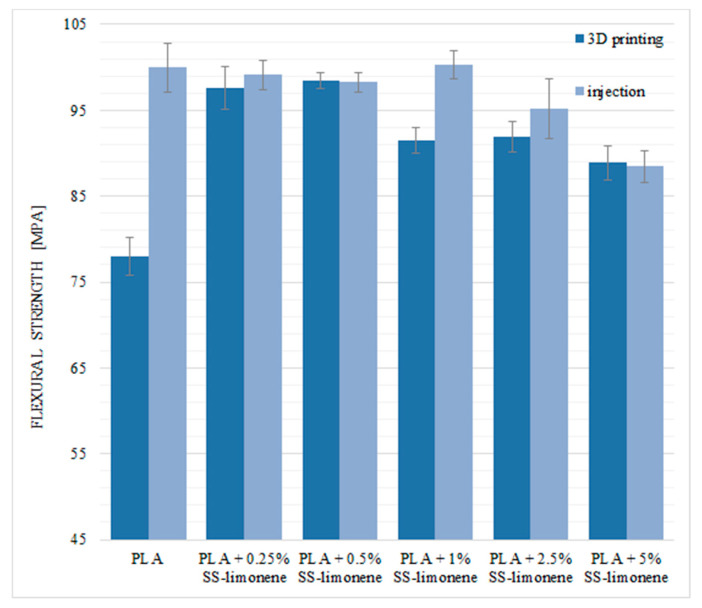
Flexural strength of SS-Limonene/PLA in 3D printing and injection moulding.

The basic flexural parameters of the samples were determined. For the 3D-printed samples, the bending load was applied perpendicularly to the plane of layer-by-layer deposition. The basic values of flexural strength for pure PLA are 77.98 MPa for the samples obtained by the FDM method and 99.98 MPa for the injection-moulded samples (Figure 10). The flexural strength is also significantly lower for the neat PLA obtained by 3D printing—this is due to the presence of air gaps and limited inter-layer adhesion, the structural discontinuities acting as stress concentration points.

In the case of the 3D printing technique, the addition of the SS-Limonene modifier significantly increased the value of the flexural strength. The samples obtained by printing with the modifier showed similar mechanical properties to those mould-injected. The highest values were obtained for the systems containing 0.25% and 0.5% of SS-Limonene (respectively 97.61 MPa; 98.46 MPa). Higher concentrations of the modifier caused a slight decrease in the strength values in relation to the 0.25% and 0.5% systems, but they were still higher than for the neat PLA samples.

The samples obtained by injection moulding technique were characterized by high values of flexural strength ranging from 88.46 MPa to 100.30 MPa (5% SS-Limonene/PLA and 1% SS-Limonene/PLA, respectively).

**Figure 11:**
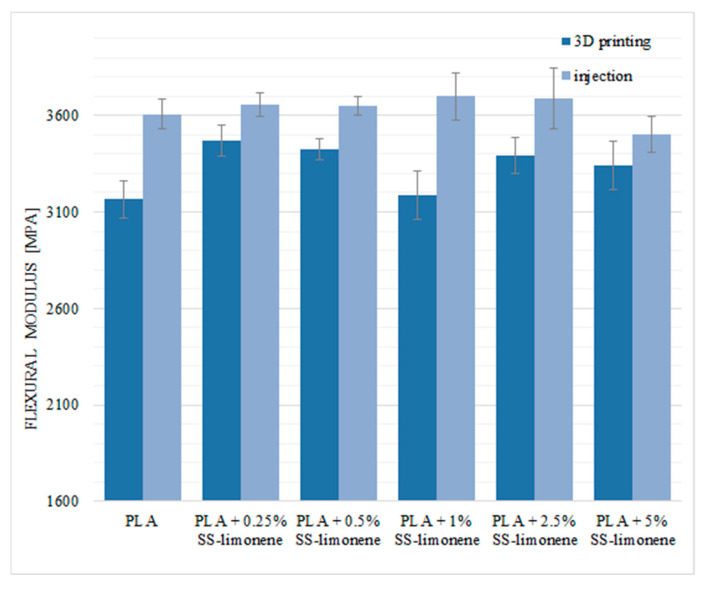
Flexural modulus of SS-Limonene/PLA in 3D printing and injection moulding.

The values of flexural modulus for both types of samples were basically unchanged regardless of the additive loading (Figure 11).

#### 2.7.3. Impact Strength and Hardness

**Figure 12:**
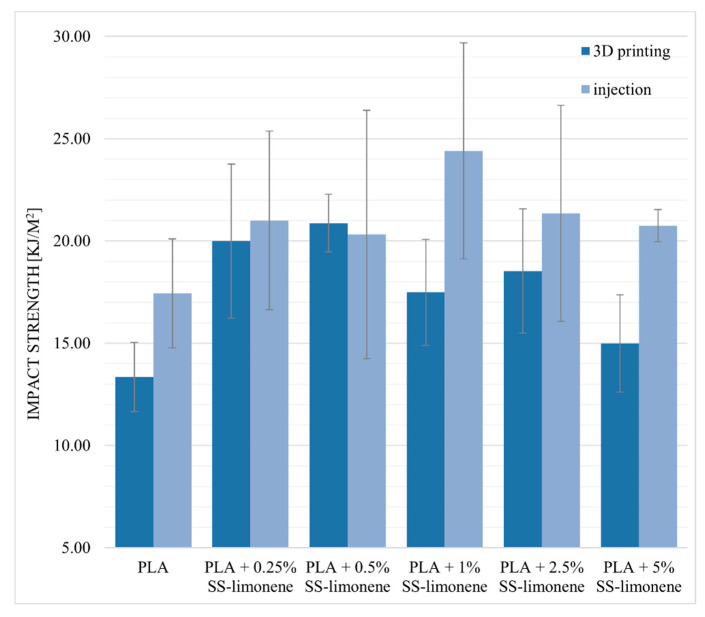
Impact strength of SS-Limonene/PLA in 3D printing and injection moulding.

Impact strength of the obtained composite samples was determined. For the 3D-printed samples, the impact direction was perpendicular to the plane of layer-by-layer deposition. Impact resistance tests confirmed the beneficial effect of SS-Limonene (especially at lower loadings) on the tested samples, regardless of the processing technique used (Figure 12). The modifier, as in the case of the previously discussed mechanical tests, acts as a plasticizer, the brittleness of the polymer is reduced therefore, the obtained composite is able to absorb more energy during an impact. High standard deviations are characteristic of the measurement method. The downward tendency along with the increase in the modifier content indicates a limited dispersion of the modifier in the polymer matrix and compatibility of the system components. Hardness, in Shore D scale, was determined to be virtually unaffected by the additive and on the level of 82–84 for all the samples regardless of the SS-Limonene loading or the processing method.

## 3. Materials and Methods

### 3.1. Materials

Polylactide (PLA) type Ingeo 2003D was purchased from NatureWorks (Minnetonka, MN, USA). The chemicals were purchased from the following sources: Tetraethoxysilane (TEOS) from Unisil (Poland), chlorodimethylsilane, tetramethylammonium hydroxide (TMAH) 25% methanol solution from ABCR, (R)-(+)-limonene, toluene, chloroform-d, Karstedt’s catalyst xylene solution from Aldrich, P_2_O_5_ from Avantor Performance Materials Poland S.A. Toluene was degassed and dried by distilling it from P_2_O_5_ under argon atmosphere.

### 3.2. Analyses

^1^H, ^13^C, and ^29^Si Nuclear Magnetic Resonance (NMR) spectra were recorded at 25 °C on a Bruker Ascend 400 and Ultra Shield 300 spectrometers using CDCl_3_ as a solvent. Chemical shifts are reported in ppm with reference to the residual solvent (CHCl_3_) peaks for ^1^H and ^13^C.

MALDI-TOF mass spectra were recorded on a UltrafleXtreme mass spectrometer (Bruker Daltonics), equipped with a SmartBeam II laser (355 nm) in the 500–4000 *m*/*z* range. 2,5-Dihydroxybenzoic acid (DHB, Bruker Daltonics, Bremen, Germany) served as matrix. Mass spectra were measured in reflection mode. The data were analysed using the software provided with the Ultraflex instrument—FlexAnalysis (version 3.4).

Fourier Transform-Infrared (FT-IR) spectra were recorded on a Nicolet iS 50 Fourier transform spectrophotometer (Thermo Fisher Scientific) equipped with a diamond ATR unit with a resolution of 0.09 cm^−1^.

Contact angle analyses were performed by the sessile drop technique at room temperature and atmospheric pressure, with a Krüss DSA100 goniometer (Hamburg, Germany). Three independent measurements were performed for each sample, each with a 5 µL water drop, and the obtained results were averaged to reduce the impact of surface nonuniformity.

Thermogravimetry (TG) was performed using a NETZSCH 209 F1 Libra gravimetric analyser (Selb, Germany). Samples of 5 ± 0.2 mg were cut from each granulate and placed in Al_2_O_3_ crucibles. Measurements were conducted under nitrogen (flow of 20 mL/min) in the range of 30–800 °C and a 20 °C/min heating rate. Differential scanning calorimetry (DSC) was performed using a NETZSCH 204 F1 Phoenix calorimeter Samples of 6 ± 0.2 mg were cut from each granulate and placed in an aluminium crucible with a punctured lid. The measurements were performed under nitrogen in the temperature range of −20–290 °C and at a 20 °C/min heating rate, and T_g_ was measured from the second heating cycle.

The effect of the modifier addition on the mass flow rate (MFR) was also determined. The measurements were made using a Instron plastometer (Norwood, MA, USA), model Ceast MF20 according to the applicable standard ISO 1133. The measurement temperature was 190 ± 0.5 °C, while the piston loading was 2.16 kg.

For flexural and tensile strength tests, the obtained materials were printed into type 1B dumbbell specimens in accordance with EN ISO 527:2012 and EN ISO 178:2006. Tests of the obtained specimens were performed on a universal testing machine INSTRON 5969 with a maximum load force of 50 kN. The traverse speed for tensile strength measurements was set at 2 mm/min, and for flexural strength was also set at 2 mm/min. Charpy impact test (with no notch) was performed on a Instron Ceast 9050 impact-machine according to ISO 179−1. For all the series, 6 measurements were performed.

Hardness of the composite samples was tested by the Shore method using a durometer Bareiss Prüfgerätebau GmbH.

A scanning electron microscope (SEM 3000, Hitachi, Japan) was used to analyse the microstructure and quality of the produced composite samples after 3D printing and injection moulding. Additionally, the effect of SS-Limonene addition on the microstructure of composite materials was investigated. Before the measurement, samples’ cross-sections were coated with a thin layer of Au-Pd. The applied voltage for SEM observations was 15 kV.

Surface structure and breakthroughs were analysed under Digital Light Microscope Keyence VHX 7000 with 100× to 1000× VH-Z100T lens (Osaka, Japan). All of the pictures were recorded with a VHX 7020 camera.

### 3.3. The Procedure for Synthesis of Octaspherosilicate Limonene Derivative (SS-Limonene)

Octahydrospherosilicate was prepared according to a literature procedure [36]. The hydrosilylation reaction was performed accordingly to a previous report [31].

In a typical procedure, a 500 mL three-neck, round-bottom flask was charged with 25 g of Octahydrospherosilicate, 250 mL of toluene and 26.77 g of limonene, and a magnetic stirring bar was added. A thermometer and condenser equipped with an argon inlet and oil bubbler were attached, the flask placed in a heating mantle and the system was purged with argon. The reaction mixture was set on 110 °C and before reaching boiling, 25 µL of Karstedt’s catalyst solution was added, which resulted in quick increase of temperature and the system starting to reflux. The reaction mixture was kept at reflux and samples were taken for FT-IR control until full Si-H group consumption was observed. Then, the solvent was evaporated under vacuum to dryness to obtain an analytically pure sample.

### 3.4. Fabrication of Filaments

#### 3.4.1. Preparation of Granulates 

The polymer and the filler were homogenized using a laboratory two-roll mill ZAMAK MERCATOR WG 150/280. A portion of 500 g PLA Ingeo™ 2003 D was mixed with SS-Limonene, until the final concentration of the additive of 5.0% *w*/*w*. The mixing was performed at the rolls temperature of 200 °C for 15 min., getting to full homogeneity of the concentrates. Masterbatch was granulated by a grinding mill WANNER C17.26 sv. The granulates were diluted with pure PLA up to the final filler concentrations of 0.25, 0.5, 1.0, 2.5 and 5.0% *w*/*w* upon extrusion moulding of a stream with consequent cold granulation on the twin-screw extrusion setup line HAAKE Rheomex OS, and then dried for 24 h at 40 °C.

#### 3.4.2. Extrusion of Filaments

The granulates obtained as above were used for moulding of filaments of 1.75 mm in diameter by a single-screw extrusion setup HAAKE Rheomex OS.

#### 3.4.3. 3D Printing (FDM)

Using a 3D printer FlashForge Finder two types of samples were printed by FDM: oars and bars, according to PN-EN-ISO 527-2. Parameters of printing are given in Table 4.

### 3.5. Injection Moulding

To compare the mechanical properties of composite materials made by 3D printing, the samples were produced by the injection moulding method. Specimens for static tensile, three-point bending, and impact tests were in accordance with the dimensions of the following standards: PN-EN ISO 527, PN-EN ISO 178, and PN-EN ISO 179, respectively. HAAKE Minijet Pro Piston Injection Moulding System (ThermoScientific, Bremen, Germany) equipped with a set of moulds was used to produce test samples. Parameters of the injection process are presented in Table 5

## 4. Conclusions

The obtained results confirm the effect of the addition of SS-Limonene on the improvement of rheological and mechanical properties of printed composites based on the PLA matrix. It is especially important for the FDM-based method for objects manufacturing, as usually objects prepared by such show much lower parameters than their injection-moulded counterparts, which can be explained by poor inter-layer adhesion and the presence of air gaps. On the basis of the obtained data, it can be concluded that the addition of a functionalized spherosilicate significantly improves such parameters as tensile strength, bending strength and impact strength of the samples obtained by 3D printing. SS-Limonene acted as a plasticizing additive for PLA. Additionally, the presence of SS-Limonene was found to increase hydrophobicity of the obtained composites. The addition of this modifier also facilitates the printing process itself, contributes to the improvement of rheological properties (reduces viscosity, increases MFR) and reduces production waste. It should be noted that in the conditions of processing the additive was found to undergo polymerization, leading to its secondary agglomeration observed under the microscope on increasing loading. It seems that the nature of the observed phenomena is complex, and the additive most likely exists in the system in two forms, namely a well-dispersed one and the agglomerated one, as suggested by previous research and the analysis of thermal data. The behaviour of the obtained composites is based on two types of interactions—between PLA and well-dispersed phase, as well as between PLA and the polymerized, agglomerated phase. Nevertheless, this complex interaction scheme should not be considered undesirable as it leads to a final improvement of the printing material system.

## Figures and Tables

**Table 1:** Masses and the numbers of models examined in order to establish waste factor values.

Sample	5%	2.5%	1%	0.5%	0.25%
**Total**					
Mass [g]	111.11	84.43	91.15	105.52	59.95
Number	38	30	30	34	24
**Correct**					
Mass [g]	53.55	51.69	57.44	66.85	44.16
Number	14	12	14	16	11
W_f_ [m%]	48	61	63	63	74
W_f_ [q%]	37	40	47	47	46

**Table 2:** Results of thermogravimetric analysis.

	1% Mass Loss [°C]		Onset Temperature [°C]		Temperature at the Maximum Rate of Mass Loss [°C]		Dry Mass Left [%]	
Conditions	N_2_	Air	N_2_	Air	N_2_	Air	N_2_	Air
SS-limonene	166.7	221.0	410.6	397.8	458.6	442.3	44.26	46.99
Neat PLA	304.8	307.9	351.1	348.3	370.1	367.0	0.00	0
PLA + 0.25% SS-Limonene	318.4	287.3	346.3	353.4	367.0	366.5	1.0	0
PLA + 0.5% SS-Limonene	316.3	308.2	348.3	346.1	368.9	366.8	0	0
PLA + 1% SS-Limonene	291.4	307.8	341.4	345.2	368.3	366.3	0.04	0
PLA + 2.5% SS-Limonene	294.9	287.9	342.4	348.5	368.4	365.5	1.03	0
PLA + 5% SS-Limonene	301.4	302.5	343.6	345.9	369.0	366.5	1.54	1.12

**Table 3:** Water contact angle [°].

	Injection Moulding [°]	3D Printing [°]
Neat PLA	83.6	81.4
PLA + 0.25% SS-Limonene	87.9	92.4
PLA + 0.5% SS-Limonene	87.8	95.4
PLA + 1% SS-Limonene	84.7	92.3
PLA + 2.5% SS-Limonene	85.7	95.1
PLA + 5% SS-Limonene	85.6	97.5

**Table 4:** Process parameters for sample printing.

Layer height	0.18 mm
Top layer height	0.27 mm
Shells	2
Top and bottom layers number	3
Bottom layers number	3
Infill density	100%
Infill pattern	Grid
Printing speed	60 mm/s
Idle speed	80 mm/s
Extruder temp.	220 °C

**Table 5:** Injection process parameters.

Cylinder temperature	225 °C
Mould temperature	45 °C
Injection pressure	750 bar
Injection time	10 s
Post-injection pressure	700 bar
Post-injection time	10 s
